# Comprehensive Surveillance of Virus Infection among Captive African Pygmy Hedgehogs in Japan

**DOI:** 10.3390/v14050857

**Published:** 2022-04-21

**Authors:** Iori Koizumi, Hina Tsukada, Daisuke Hayasaka, Hiroshi Shimoda

**Affiliations:** 1 Koizumi Nest Animal Hospital, 3-24 Bettou, Yahata Nishi, Kitakyushu, Fukuoka 806-0062, Japan; mail@koizumi-nest.com; 2 Laboratory of Veterinary Microbiology, Joint Graduate School of Veterinary Science, Yamaguchi University, 1677-1 Yoshida, Yamaguchi 753-8515, Japan; dhaya@yamaguchi-u.ac.jp; 3 Laboratory of Veterinary Microbiology, Joint Faculty of Veterinary Medicine, Yamaguchi University, 1677-1 Yoshida, Yamaguchi 753-8515, Japan; htsuka1206@gmail.com

**Keywords:** African pygmy hedgehog, adenovirus, herpesvirus

## Abstract

African pygmy hedgehogs (*Atelerix albiventris*) are popular exotic pets in Japan, and their breeding numbers have recently increased. Although various diseases have been reported in hedgehogs, including skin, respiratory, neurological, and neoplastic diseases, most of the causes remain unidentified. In this study, we investigated herpesvirus, adenovirus, and coronavirus infections among 150 African pygmy hedgehogs in Japan and evaluated the correlations between virus infection and diseases. A novel herpesvirus named Atelerix albiventris herpesvirus 1 (AAHeV), and African pygmy hedgehog adenovirus 1 (AhAdV-1) were detected in 14 and 3 oral swab samples, respectively. AAHeV infection may be related to neurological clinical signs. Interestingly, no hedgehog with a neoplastic disorder tested positive for AAHeV. Further research is required to determine the pathogenicity and prevalence of the detected viruses.

## 1. Introduction

A hedgehog is a small, spiny insectivore that has become popular in recent years as an exotic pet in Japan. Among the 16 hedgehog species, African pygmy hedgehog (*Atelerix albiventris*) is bred as a companion animal. With the increasing number of pet hedgehogs in Japan, various diseases have been reported in hedgehogs, including skin, respiratory, neurological, and neoplastic diseases [1].

Although the causes of these diseases are not well understood, previous reports have suggested that viral infections may be a contributing factor. For example, African pygmy hedgehog adenovirus 1 (AhAdV-1) was detected from the throat/nasal swab of African pygmy hedgehogs that showed respiratory clinical signs and died in a colony in Japan [2]. The pathological diagnoses of the dead hedgehogs included respiratory disorders, such as bronchitis and pneumonia.

Herpesvirus infection has been reported in African pygmy hedgehogs and European hedgehogs (*Erinaceus europaeus*). Examinations of European hedgehogs that died at the Newcastle Veterinary Investigation Centre in the United Kingdom revealed histological changes in the liver and amphophilic inclusion bodies with virus particles in hepatocytes [3]. Widén et al. also reported that the cultivation of the liver homogenate from a young European hedgehog that died led to alpha herpesvirus-like cytopathic effects (CPE) with intranuclear inclusion bodies [4]. Human herpes simplex virus was also isolated from an African pygmy hedgehog that showed neurological clinical signs with posterior ataxia and fast progressing paresis. Postmortem examinations revealed randomly distributed foci of necrosis in the liver, and human herpes simplex virus antigens were detected in neurons and some glial cells using immunolabeling [5].

Coronavirus was detected in the fecal samples of free-living European hedgehogs in several European countries, including Germany [6], France [7], and the United Kingdom [8,9], as well as in Amur hedgehogs from China [10]. In these reports, no infectious viruses were isolated, and the animals did not show any clinical signs associated with coronavirus infection.

Pneumonia virus of mice (PVM) is suspected to be linked to wobbly hedgehog syndrome. Madarame et al. detected the PVM RNA genome using next-generation sequencing, and PVM antigen using immunohistochemical analysis in the brain of an African pygmy hedgehog that showed encephalitis in Japan [11].

Taken together, these reports indicate that hedgehogs have the potential to become reservoirs for these pathogens, including zoonotic viruses. Therefore, surveys of infectious agents in Japanese hedgehogs are expected to provide useful information for the prevention of emerging infectious diseases not only in hedgehogs, but also in humans. In the present study, we investigated the viral infections among African pygmy hedgehogs in Japan with a focus on herpesvirus, adenovirus and coronavirus, and assessed the correlations between virus infection and various diseases.

## 2. Materials and Methods

### 2.1. Sample Collection

Oral swabs were collected from 150 domestic hedgehogs at a veterinary hospital between March 2019 and June 2020. Most of them presented some clinical signs. Swab samples were mixed in a vortex mixer in sterilized phosphate-buffered saline and stored at −80 °C until examination.

### 2.2. Detection of Herpesvirus, Adenovirus, and Coronavirus from Hedgehog Swab Samples

DNA extraction from swab samples was conducted using the DNeasy Blood & Tissue Kit (QIAGEN, Hilden, Germany) according to the manufacturer’s instructions. Nested polymerase chain reaction (PCR) assays were performed using the TaKaRa Ex Taq kit (TaKaRa, Otsu, Japan) for the detection of herpesvirus and adenovirus. Pan-herpesvirus primers were used for the detection of the herpesvirus DNA polymerase gene (215 to 315 bp in length), as described previously [12] (Table 1). The first and second rounds of the reaction consisted of an initial denaturation at 94 °C for 5 min, followed by 45 cycles of denaturation at 94 °C for 30 s, annealing at 46 °C for 30 s and extension at 72 °C for 1 min, with a final extension at 72 °C for 5 min. Pan-adenovirus primers were used for the detection of the adenovirus DNA polymerase gene (318 to 324 bp in length), as described previously [13] (Table 1). The first and second rounds of the reaction consisted of an initial denaturation at 94 °C for 2 min, followed by 45 cycles of denaturation at 94 °C for 30 s, annealing at 46 °C for 1 min and extension at 72 °C for 1 min, with a final extension at 72 °C for 7 min. RNA was extracted from swab samples using a Viral RNA Mini Kit (QIAGEN, Hilden, Germany) according to the manufacturer’s instructions. Reverse transcription (RT)-PCR was performed using a QIAGEN OneStep RT-PCR Kit (QIAGEN, Hilden, Germany) and pan-coronavirus primers targeting the coronavirus RNA-dependent RNA polymerase (440 bp), as described previously [14] (Table 1). The RT-PCR conditions were as follows: an initial step of 30 min at 50 °C for reverse transcription; 15 min at 95 °C for denaturation; 40 cycles of denaturation at 94 °C for 1 min, annealing at 48 °C for 1 min and extension at 72 °C for 1 min; and a final extension step at 72 °C for 10 min. All amplicons were visualized by gel electrophoresis on 2% agarose gels.

### 2.3. Phylogenetic Analysis

Sequence analysis was performed using the MEGA 10.0 software [15]. The primer-trimmed sequences detected in this study were aligned by ClustalW and compared to previously reported herpesvirus or adenovirus sequences in GenBank using the Basic Local Alignment Search Tool (BLAST). Neighbor-joining trees were constructed using the MEGA 10.0 software with a bootstrap analysis of 1000 replicates and *p*-distance models.

### 2.4. Specific PCR for the Detected Viruses

Specific nested PCR for the detected viruses was performed with the TaKaRa Ex Taq kit using specific primer pairs constructed based on the sequence data (Table 1). The first and second PCR reactions consisted of an initial denaturation at 94 °C for 2 min, followed by 45 cycles of denaturation at 94 °C for 30 s, annealing at 50 °C for 1 min in the first round and at 55 °C for 1 min in the second round, extension at 72 °C for 1 min, and a final extension at 72 °C for 7 min. The second-round amplicons were visualized by gel electrophoresis on 2% agarose gels.

### 2.5. Virus Isolation

MDCK cells (JCRB9020; Japanese Collection of Research Bioresources Cell Bank, Osaka, Japan) and BHK-21 cells (JCRB9029; Japanese Collection of Research Bioresources Cell Bank, Osaka, Japan) were used for virus isolation. The cells were cultured in Dulbecco’s Modified Eagle Medium (Thermo Fisher Scientific, Waltham, MA, USA) containing 10% heat-inactivated fetal calf serum, 100 U/mL penicillin, and 100 μg/mL streptomycin (Thermo Fisher Scientific, Waltham, MA, USA). The cells were maintained at 37 °C in 5% CO_2_. Virus isolation was performed by inoculating filtrated swab suspensions onto monolayers of MDCK and BHK-21 cells. The cells were incubated at 37 °C in 5% CO_2_ and observed daily for CPE. The cells were blind-passaged five times until CPE were seen.

### 2.6. Statistical Analysis

Molecular epidemiological data were analyzed according to sex (male and female), age class (juvenile and adult), and clinical signs (respiratory disorder, digestive disorder, oral disease, neurological disease, skin disease, and neoplastic disorder) using the chi-square test. *p* values < 0.05 were considered to be statistically significant.

## 3. Results

### 3.1. Detection of Herpesvirus and Adenovirus from Oral Swab Samples of Hedgehogs

**Figure 1:**
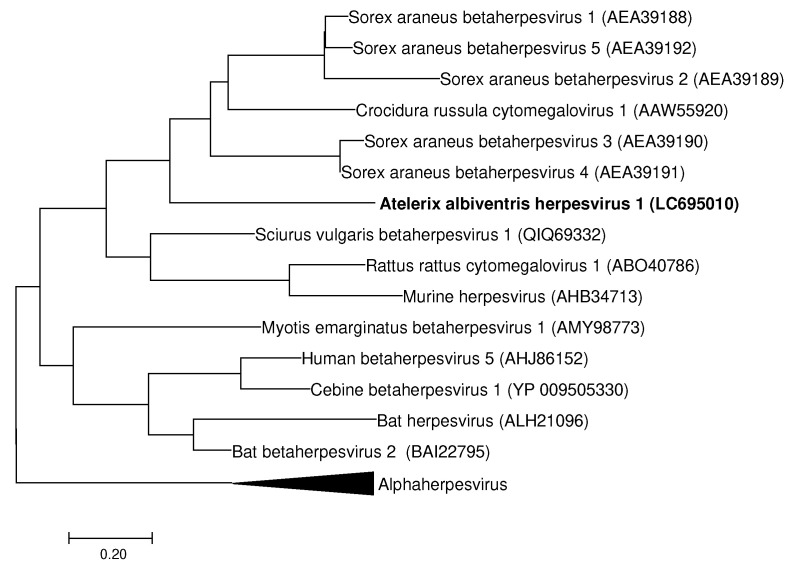
Phylogenetic tree of the detected herpesviruses based on the partial amino acid sequences of DNA polymerase. This phylogenetic tree was constructed based on 49 amino acids of the DNA polymerase gene. Sequences of the novel herpesvirus detected in this study are shown in bold. GenBank accession numbers of the listed viruses are shown in parentheses.

**Figure 2:**
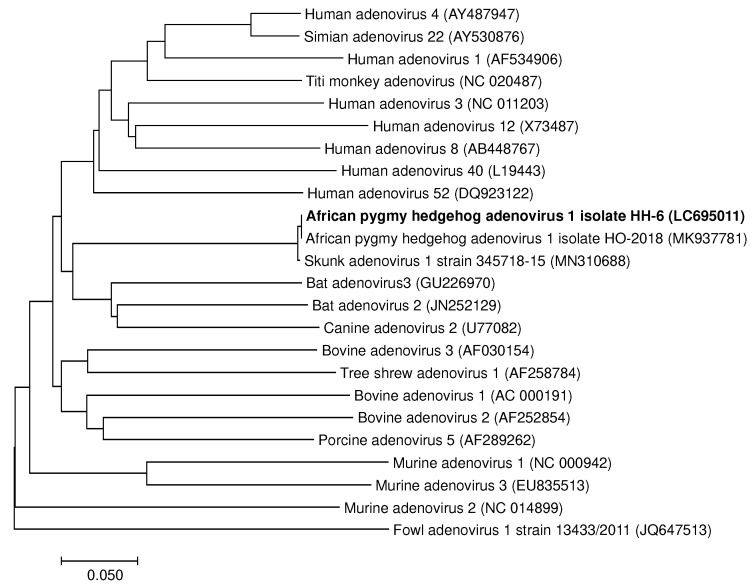
Phylogenetic tree of the detected adenoviruses based on the partial amino acid sequences of DNA polymerase. This phylogenetic tree was constructed based on 91 amino acids of the DNA polymerase gene. Sequences of the adenovirus detected in this study are shown in bold. GenBank accession numbers of the listed viruses are shown in parentheses.

Oral swab samples were collected from 150 hedgehogs with various health conditions that were taken to a veterinary clinic in Fukuoka, Japan, between March 2019 and June 2020. First, 50 of the 150 oral swab samples were analyzed for herpesvirus, adenovirus, and coronavirus using universal primer pairs, in accordance with previous studies [12,13,14]. Among the 50 analyzed samples, 2 were positive for herpesvirus, 2 were positive for adenovirus, and 0 were positive for coronavirus (Table 2). Based on sequence analysis, the virus in both of the herpesvirus-positive samples was identified to be a novel betaherpesvirus named Atelerix albiventris herpesvirus 1 (AAHeV, GenBank accession number LC695010). Among viruses with a query coverage of over 80%, AAHeV showed the highest amino acid similarity (with 46% identity and 94% query coverage) to Sorex araneus betaherpesvirus 4 (GenBank accession number AEA39191), which was isolated from a common shrew (Figure 1). In addition, in the two adenovirus-positive samples (GenBank accession number LC695010), the virus was 100% identical to African pygmy hedgehog adenovirus 1 (AhAdV-1, GenBank accession number MK937781, Figure 2), which was isolated from hedgehogs that showed respiratory clinical signs in Japan [16].

### 3.2. Molecular Epidemiology of the Detected Viruses

For further epidemiological study, we analyzed all 150 swab samples taken from the hedgehogs of the same hospital, including the previously examined 50 samples, using specific primers for AAHeV and AhAdV-1. Of the 150 samples, 14 (9.3%) and 3 (2.0%) were positive for AAHeV and AhAdV-1, respectively (Table 2). The sequences of all the partial genomes detected by molecular epidemiology were 100% identical to AAHeV (GenBank accession number LC706228-706240) or AhAdV-1 (GenBank accession number LC706226-706227) detected in the first screening. The data above were further analyzed according to sex (male and female), age class (juvenile and adult), and clinical signs (respiratory disorder, digestive disorder, oral disease, neurological disease, skin disease, and neoplastic disorder). The prevalence of AAHeV was significantly higher in hedgehogs with neurological clinical signs (*p* = 0.016; Table 3). Interestingly, there were no AAHeV-positive hedgehogs among the 49 hedgehogs with a neoplastic disorder (Table 3). The prevalence of AAHeV was not significantly related to sex, age class, respiratory disorders, digestive disorders, oral diseases, or skin diseases (Table 3). However, the data for AhAdV-1 may not be reliable due to the small number of positive samples. While there was no significant difference according to sex, the AhAdV-1-positive rate was significantly higher in juvenile hedgehogs than in adult hedgehogs (*p* < 0.0004; Table 3). The AhAdV-1-positive rate was significantly higher in hedgehogs with a respiratory disorder (*p* < 0.045; Table 3), while the other clinical signs showed no significant correlation. The prevalence of AhAdV-1 was not significantly related to sex, neurological diseases, neoplastic disorders, digestive disorders, oral diseases, or skin diseases (Table 3).

Unfortunately, although we attempted to isolate viruses from the AhAdV-1-positive samples using BHK-21 and MDCK cells, no CPE were observed, and no viral DNA was detected in the inoculated cells (data not shown).

## 4. Discussion

The results of the present study indicated that a novel herpesvirus, AAHeV, and AhAdV-1 are infecting African pygmy hedgehogs bred in Japan with a prevalence of 9.3% and 2.0%, respectively.

Although alpha and gamma herpesvirus infections have been reported in hedgehogs previously [17], this is the first report of a betaherpesvirus infecting hedgehogs. Since we only examined a partial sequence of the DNA polymerase gene of AAHeV, additional sequences and/or virus isolation are required for further characterization of the detected virus, including its pathogenicity, transmission cycle, and growth kinetics. Although there has been no report on the isolation of hedgehog herpesvirus, the isolation of human herpesvirus from hedgehog samples has been described [4,5]. The establishment of optimal cultured cell lines derived from hedgehogs would provide useful tools for hedgehog herpesvirus isolation.

Our study results indicated a significant relationship between AAHeV infection and neurological diseases. A major neurological disease in hedgehogs is wobbly hedgehog syndrome, but its cause has not yet been clarified. Various factors have been suspected to cause the disease, including genetic abnormality [18], malnutrition, stress, and viruses [11]. AAHeV infection may be an important factor in the development of neurological diseases in hedgehogs.

It is also notable that none of the hedgehogs with a neoplastic disease were positive for AAHeV infection. Although there has been a report on the detection of a retrovirus in hedgehog sarcomas [19], the cause of hedgehog tumors remains unclear. Interestingly, herpes simplex virus type 1, belonging to *Alphaherpesvirinae*, is a well-known oncolytic virus, and it has been studied for potential application as a therapeutic approach for cancer [20]. Oncolytic viruses replicate in tumor cells, selectively kill the tumor cells [21], and induce anti-tumor immune responses [22]. If AAHeV infection has potential effects on the host’s response to oncogenicity, further research is necessary to elucidate the developmental mechanisms of tumors in hedgehogs.

In the present study, one of the three AhAdV-1-positive hedgehogs had a respiratory disorder. Although the statistical analysis results suggested a correlation between respiratory signs and AhAdV-1 infection, the low number of positive-testing hedgehogs and the incidental respiratory disease should be carefully taken into consideration. Recent surveillance data have suggested that AhAdV-1 [2] and skunk adenovirus 1 [23], which is closely related to AhAdV-1, may be the dominant respiratory pathogens among hedgehogs. In particular, skunk adenovirus 1 was reported to have caused fatal bronchopneumonia in an African pygmy hedgehog [23], suggesting that AhAdV-1 may also cause fatal respiratory diseases in hedgehogs. However, since AhAdV-1 was detected in hedgehogs with no respiratory diseases, subclinical infection of AhAdV-1 may occur among hedgehogs. Further analyses with a larger number of cases are required to gain more convincing data on the relationship between respiratory diseases and AhAdV-1 infection in hedgehogs.

In conclusion, the results of the present study indicated that a novel betaherpesvirus and AhAdV-1 are infecting African pygmy hedgehogs bred in Japan. The results suggest that AAHeV infection may play a key role in the mechanism of neurological and neoplastic diseases in hedgehogs, but further studies are needed to better assess the relationship between AhAdV-1 and diseases in hedgehogs. Additional investigations are also required to determine the pathogenicity and prevalence of these viruses in hedgehogs.

## Figures and Tables

**Table 1:** Primers used in this study.

Target Virus	Primer Name *	Primer Sequence (5′->3′)	Product Size (bp)	Reference
Herpesvirus (universal)	DFA (1st)	GAYTTYGCNAGYYTNTAYCC	215–315	[12]
	ILK (1st)	TCCTGGACAAGCAGCARNYSGCNMTNAA		
	KG1 (1st)	GTCTTGCTCACCAGNTCNACNCCYTT		
	IYG (2nd)	CACAGAGTCCGTRTCNCCRTADAT		
	TGV (2nd)	TGTAACTCGGTGTAYGGNTTYACNGGNGT	168	
African pygmy hedgehog herpesvirus	HHHeV_3F (1st)	GTTACCTTGTTTGCCTGTGGC		This study
	HHHeV_9F (2nd)	GCTTCGGTGACGAAAATCGG		
	HHHeV_9R (1st, 2nd)	TTCATCGTTTGTCTCTGTGGT		
Adenovirus (universal)	polFouter (1st)	TNMGNGGNGGNMGNTGYTAYCC	318–324	[13]
	polRouter (1st)	GTDGCRAANSHNCCRTABARNGMRTT		
	polFinner(2nd)	GTNTWYGAYATHTGYGGHATGTAYGC		
	polRinner (2nd)	CCANCCBCDRTTRTGNARNGTRA		
African pygmy hedgehog adenovirus	AhAdV-pol-1523F (1st)	CTGGCATACATCCCGCARAT	287	This study
	AhAdV-pol-1976R (1st)	CAGATGGGTTTCCCGCTCTT		
	AhAdV-pol-1601F (2nd)	CCTCGGATACTGGACCTGAC		
	AhAdV-pol-1887R (2nd)	TACGACATCATCCAGCACACC		
Coronavirus (universal)	IN-6	GGTTGGGACTATCCTAAGTGTGA	440	[14]
	IN-7	CCATCATCACATAGAATCATCAT		

* In parentheses indicate whether the primer was used in 1st or 2nd PCR.

**Table 2:** Prevalence of virus infection among hedgehogs in Japan.

	Universal Primer			Specific Primer	
	Herpesvirus	Adenovirus	Coronavirus	HHHeV	AhAdV-1
No. of tested samples	50	50	50	150	150
No. of positive samples	2	2	0	14	3
% of positive samples	4%	4%	0%	9.3%	2.0%

**Table 3:** Comparison of virus infection and status of hedgehogs.

Characteristic	Status	No. of Tested Samples	No. of Positive Samples *		*p* Value	
			AAHeV	AhAdV	AAHeV	AhAdV
sex	Male	83	8 (10%)	0 (0%)	0.813	0.091
	Female	59	5 (8%)	2 (3%)		
Age class	Juvenile (<6 months)	13	1 (8%)	2 (15%)	0.796	0.0004
	Adult (≥6 months)	131	13 (9.9%)	1 (0.8%)		
Neurological disease	Yes	27	6 (22%)	0 (0%)	0.016	0.410
	No	122	8 (6.6%)	3 (2.5%)		
Neoplastic disease	Yes	49	0 (0%)	1 (2%)	0.006	0.987
	No	100	14 (14.0%)	2 (2.0%)		
Respiratory disease	Yes	9	1 (11%)	1 (11%)	0.856	0.045
	No	140	13 (9.3%)	2 (1.4%)		
Digestive disease	Yes	17	2 (12%)	1 (6%)	0.730	0.231
	No	131	12 (9.2%)	2 (1.5%)		
Oral disease	Yes	33	3 (9%)	0 (0%)	0.957	0.353
	No	117	11 (9.4%)	3 (2.6%)		
Skin disease	Yes	40	4 (10%)	1 (3%)	0.878	0.798
	No	109	10 (9.2%)	2 (1.8%)		

* In parentheses indicate the percentage of positive samples.

## Data Availability

Not applicable.
